# Spontaneous gastrocnemius muscle haematoma formation in a patient with alcoholic liver disease

**DOI:** 10.11604/pamj.2020.36.348.24623

**Published:** 2020-08-26

**Authors:** Ndawapeka Tulonga Nhinda, Innocent Lule Segamwenge, Twitileni Nampweya, Chrizelda Engels

**Affiliations:** 1 Department of Internal Medicine, Intermediate Hospital Oshakati, Oshana, Namibia,; 2 School of Medicine, University of Namibia, Windhoek Namibia

**Keywords:** Alcoholic hepatitis, liver cirrhosis, muscle haematoma

## Abstract

Advanced liver disease is associated with several haemostatic alterations which can lead to either thrombosis or bleeding complications. Spontaneous muscle haematomas although a rare complication of liver cirrhosis is increasingly being described in the literature and hyperfibrinolysis is an emerging plausible pathophysiological mechanism. We describe a patient who presented with a life threatening spontaneous haematoma in his gastrocnemius muscle that required treatment with antifibrinolytic therapy to control further bleeding.

## Introduction

Liver cirrhosis is associated with hemostatic alterations that can lead to thrombosis as well as to bleeding complications [1]. Patients with advanced liver disease are therefore at increased risk for both bleeding and thrombotic complications. Several pathophysiological mechanisms have been advanced to explain the increased bleeding risk in advanced liver disease. These include decreased synthesis and clearance of several coagulation factors, thrombocytopenia, variceal bleeding and platelet dysfunction [2]. Increasingly, primary hyperfibrinolysis has been advanced as a cause of intramuscular haematomas in advanced liver disease [3-5]. Bleeding in advanced liver disease is common and can be due to gastroesophageal variceal bleeding and non-variceal bleeding. Non-variceal bleeding can be due to peptic ulcer bleeding, portal hypertensive gastropathy and colopathy [1,6,7]. These patients can also frequently present with minor bleeding related to primary haemostatic defects in the form of epistaxis, gum bleeding and purpuric skin lesions [1]. Bleeding related to coagulation defects is rare in advanced chronic liver disease. However, there is a growing body of evidence in the form of case reports describing spontaneous muscle haemorrhage, a fatal complication of liver cirrhosis [4,8-10]. We herewith present a case report on a spontaneous muscle haematoma in a patient with alcoholic liver disease.

## Patient and observation

**Figure 1:**
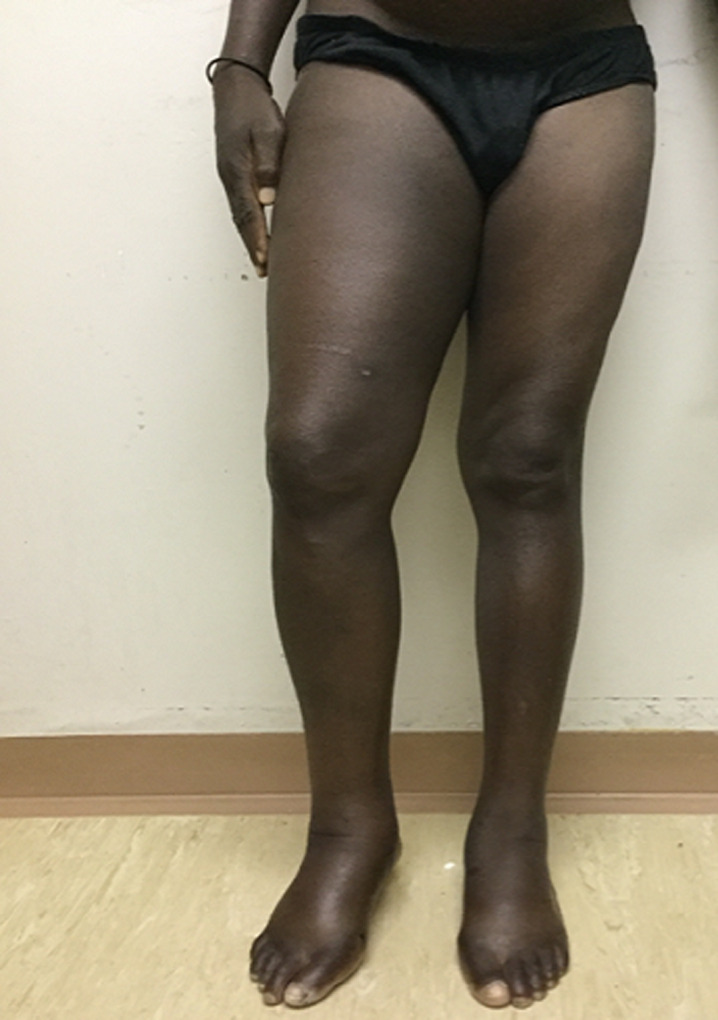
patient's leg showing swelling due to underlying gastrocnemius hematoma

A 39-year-old Namibian male was referred to Intermediate Hospital Oshakati from his local community hospital for evaluation of progressive swelling of his right thigh. The patient, with a known history of alcoholic liver disease, had been fairly well until 2 weeks prior to presentation to his local hospital, when he gradually developed jaundice which was followed by acute onset right thigh pain and swelling. He described the pain as burning in nature which radiated downwards into his right foot. The pain was exacerbated by movement and alleviated with rest. The pain gradually worsened with inability to use the right lower limb. There was associated bluish discolouration of the overlying skin. The patient denied a history of previous trauma or penetrating injury, fever, warmth to touch of the limb, loss of weight or bleeding from other orifices. Neither did the patient consume any anticoagulation therapy, NSAIDS and aspirin, nor did the patient have a previous background of a bleeding disorder or family history of a bleeding disorder. This was the 2^nd^ episode with a similar episode 4 years ago in 2015 which resolved gradually, the same year he was diagnosed with alcoholic liver disease. The rest of the medical and surgical history was unremarkable. The patient had a significant history of alcohol, drinking approximately 1 to 2 bottles of Whiskey a day during the 9 months prior to this presentation. This followed a period of alcohol cessation after the prior presentation in 2015. At presentation, he had normal blood pressure of 121/68mmHg with a tachycardic of 121beats per minute and a respiratory rate of 18 breaths/ min. He was apyrexial with a temperature of 36.5°C. He was awake and oriented without signs of hepatic encephalopathy and had scleral icterus and leukonychia. Other peripheral stigmata of chronic liver disease were absent. His right thigh had pitting oedema extending from the right foot to his gluteus (Figure 1). A circumferential difference of 11 cm measured around the mid- thigh, and 3 cm measured around the calf, compared to the left limb was present. Ecchymosis was present on the posterior thigh, which extended from the gluteal fold to the popliteal fossa. The thigh was severely tender on palpation with decreased range of motion on both active and passive movement. All distal pulses were present. The abdomen was distended with evidence of ascites, liver span of 4cm and no splenomegaly. The examination findings of other systems were normal.

**Figure 2:**
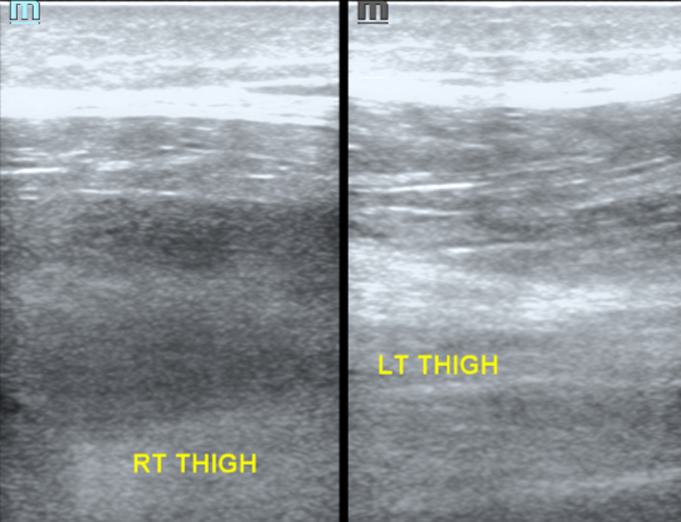
haematoma with right and left comparison

**Figure 3:**
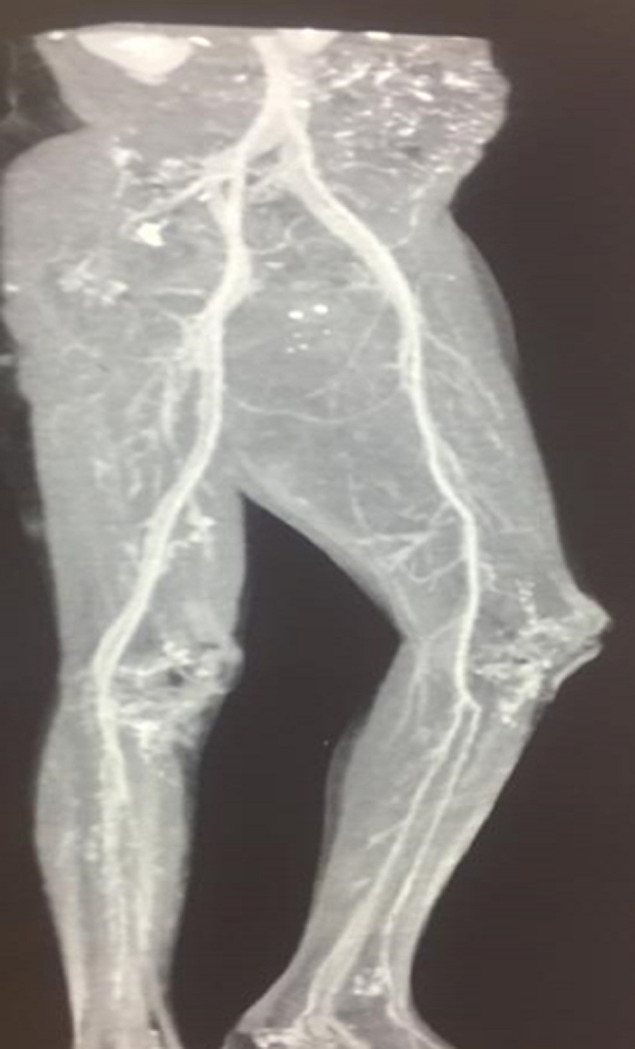
computed tomography angiogram of the distal extremities

The Investigations performed included a complete blood count which showed severe Macrocytic normochromic anaemia with a haemoglobin of 3.60g/L, in addition to thrombocytopenia with a platelet count of 37 x 10^9^/L and a reticulocyte production index of 1.1%. The international normalised ratio (INR) was 2,68, a prothrombin time (PT) of 31.30 seconds and the partial thromboplastin time (PTT) was 35.2 seconds. His liver panel showed total protein of 65g/L with hypoalbuminemia of 17g/L. The Total Bilirubin was elevated at 149 umol/L and Direct Bilirubin 48 umol/L. Alanine aminotransferase (ALT) at 19 IU/L and aspartate aminotransferase (AST) at 90 IU/L with AST to ALT ratio 4.7suggestive of Alcoholic hepatitis. Lactate dehydrogenase (LDH) was elevated at 506 IU/L gamma-glutamyl transferase (GGT) 57 IU/L, C- reactive protein 7.8 mg/l and total white cell count of 10.39x10^9^/L. The patient had a normal urea and creatinine with a hyponatraemia of 128mmol/L. His Iron studies performed revealed Ferritin at 2321.03 ng/ml with serum iron of 6.9 umol/L. His viral screen for hepatitis B and C was negative. A doppler ultrasound illustrated extensive tissue oedema with complex mass extending from the upper thigh to the lower thigh suggestive of a right gastrocnemius haematoma (Figure 2), no thrombus noted in the venous system. The ultrasound of the abdomen showed ascites with an echogenic small liver. The computer tomography angiogram (CT-angio) showed normal non-aneurysmal common iliac, femoral and popliteal arterial vessels (Figure 3). Unfortunately, upper gastrointestinal endoscopy was not performed due to local resource constraints. The patient was managed with tranexamic acid 1g eight hourly for a period of 7 days, red cell concentrate (RCC) and 3 units of fresh frozen plasma (FFP) transfusions which were repeated over 3 separate days during hospitalization as well vitamin k 10mg titrated against his serial INR measurements. He was given adequate pain relief and thiamine. He was hospitalized for 17 days, during this period the swelling gradually reduced and then maintained a constant size. Health education on his condition and prognosis was explained at which he requested to be transferred to his local hospital for better family support. He was advised to stop alcohol consumption and follow up with a local social worker for long-term alcohol support.

## Discussion

It is known that muscle haematomas can occur spontaneously in patients with haemophilia and in those who are receiving anticoagulant therapy, with the most common sites being the rectus abdominis muscle and the iliopsoas muscle [9,10]. There is a growing body of evidence describing intramuscular bleeding in patients with liver cirrhosis [4,8-12]. In these cases, the major risk factor for liver cirrhosis was alcohol and all the patients had poor outcomes. Our patient was diagnosed with a haematoma of the right gastrocnemius muscle secondary to liver cirrhosis diagnosed clinically and alcohol was the identified risk factor for the development of his liver cirrhosis. In addition, despite the liver disease, the patient was still actively consuming alcohol on a daily basis. It appears that liver disease together with long- term alcohol consumption synergistically favour a bleeding state. Alcohol abuse is thought to directly induce anti-haemostatic effects and indirectly by further aggravating coagulopathy caused by the liver [8].

Chronic liver disease affects all the three haemostasis processes i.e. primary haemostasis, coagulation, and fibrinolysis. Primary haemostasis is affected through the decrease in platelet number and function. Thrombocytopenia occurs in up to 76% of patients with cirrhotic liver disease [2]. The cause of thrombocytopenia in liver disease is multifactorial [13]. Although our patient had a platelet count of 37 x 10^9^/L, he had no evidence of bleeding related to defects in the primary haemostasis. The bleeding risk in our patient was assessed by performing a prothrombin time (PT), international normalized ration (INR) and activated partial thromboplastin time (APTT), which were all raised. In chronic liver disease, coagulation is affected by decreased production and clearance of both procoagulant and anticoagulant factors leading to what some authors have described as a balanced haemostasis. These patients therefore are at high risk of developing either thrombosis or bleeding depending on provoking circumstantial risk factors [1,2,5]. The traditionally used coagulation tests (PT) and (aPTT) may not represent the true derangement in haemostasis in liver disease and may not reliably predict the risk of bleeding [2]. Fibrinolysis is also affected in chronic liver disease through the reduced hepatic production and clearance of both profibrinolytic and antifibrinolytic proteins. There is decreased production of alpha-2 antiplasmin and thrombin activatable fibrinolysis inhibitor (TAFI), whereas the level of tissue plasminogen activator (t-PA) and plasminogen activator inhibitor-1 (PAI-1) are increased due to decreased hepatic clearance of these factors [5]. The ratio of t-PA to PAI-1 is however elevated due to the duo production of t-PA by both hepatic and endothelial cells. This balance of haemostasis leading to higher t-PA levels compared to PAI-1 is likely to favour fibrinolysis with a potential to haemorrhage. Nair and colleagues described a patient with liver cirrhosis who presented with spontaneous intramuscular hematoma which they attributed to hyperfibrinolysis [4]. They demonstrated low levels of alpha-2 antiplasmin and a decreased clot lysis time by measuring euglobulin lysis time. We were unable to measure markers of hyperfibrinolysis in our patient as these tests are not routinely available in our practice setting.

The treatment of bleeding related to hyperfibrinolysis in chronic liver disease is supportive and largely includes blood product transfusions and use of antifibrinolytic agents [4]. We treated our patient with tranexamic acid in addition to blood and fresh frozen plasma transfusions. There is a growing number of reports describing successful treatment of bleeding in liver cirrhosis with the use of antifibrinolytic agents. These agents prevent plasminogen from binding to fibrin and reduce the conversion of plasminogen to plasmin [3]. Gunawan and colleagues treated 60 cirrhotic patients with shortened euglobin lysis time using epsilon-aminocaproic acid and achieved 92% improvement in bleeding [14]. Laskiewicz and colleagues successfully treated a patient with advanced liver disease with refractory bleeding who had failed to respond to multiple transfusion products but responded to the administration of tranexamic acid, another antifibrinolytic agent [15]. Similarly, Kodali and colleagues successfully treated a patient with advanced cirrhosis who presented with spontaneous subdural hematoma who after craniotomy failed to respond to multiple transfusion products but successfully responded to the administration of tranexamic acid [16]. Our patient successfully responded to the blood products transfusion and tranexamic acid was discharged after 17 days of hospitalization. He demised later from hepatic encephalopathy on his 3^rd^ readmission. The mortality rate in these patients is high especially those in whom chronic liver disease was caused by alcohol [10,12,17]. It is important therefore to make a prompt diagnosis and initiate the appropriate management. Liver transplantation following sustained abstinence from alcohol is the only definitive treatment for patients with advanced liver disease, without which most patients have a terminal course.

## Conclusion

Spontaneous gastrocnemius muscle haematoma is a rare complication of alcoholic liver disease and hyperfibrinolysis is a common underlying aetiology. We successfully diagnosed and treated our patient with tranexamic acid, an antifibrinolytic agent to prevent life threatening bleeding.
